# Severe bacterial infections in people who inject drugs: the role of injection-related tissue damage

**DOI:** 10.1186/s12954-022-00624-6

**Published:** 2022-05-02

**Authors:** Alexander Hrycko, Pedro Mateu-Gelabert, Courtney Ciervo, Rebecca Linn-Walton, Benjamin Eckhardt

**Affiliations:** 1 Division of Infectious Diseases and Immunology, New York University School of Medicine, 462 1st Avenue, NBV 16S10, New York, NY 10016 USA; 2 Institute for Implementation Science in Population Health, City University of New York Graduate School of Public Health and Health Policy, 55 West 125th Street, Room 625, New York, NY 10027 USA; 3 Office of Behavioral Health, NYC Health and Hospitals, 125 Worth Street, Room 423, New York, NY 10013 USA

**Keywords:** Injection drug use, Severe bacterial infection, Risk factors, Tissue damage

## Abstract

**Background:**

In the context of the current U.S. injection drug use epidemic, targeted public health harm reduction strategies have traditionally focused on overdose prevention and reducing transmission of blood-borne viral infections. Severe bacterial infections (SBI) associated with intravenous drug use have been increasing in frequency in the U.S. over the last decade. This qualitative study aims to identify the risk factors associated with SBI in hospitalized individuals with recent injection drug use.

**Methods:**

Qualitative analysis (*n* = 15) was performed using an in-depth, semi-structured interview of participants admitted to Bellevue Hospital, NYC, with SBI and recent history of injection drug use. Participants were identified through a referral from either the Infectious Diseases or Addition Medicine consultative services. Interviews were transcribed, descriptively coded, and analyzed for key themes.

**Results:**

Participants reported a basic understanding of prevention of blood-borne viral transmission but limited understanding of SBI risk. Participants described engagement in high risk injection behaviors prior to hospitalization with SBI. These practices included polysubstance use, repetitive tissue damage, nonsterile drug diluting water and multipurpose use of water container, lack of hand and skin hygiene, re-use of injection equipment, network sharing, and structural factors leading to an unstable drug injection environment. Qualitative analysis led to the proposal of an Ecosocial understanding of SBI risk, detailing the multi-level interplay between individuals and their social and physical environments in producing risk for negative health outcomes.

**Conclusions:**

Structural factors and injection drug use networks directly impact drug use, injection drug use practices, and harm reduction knowledge, ultimately resulting in tissue damage and inoculation of bacteria into the host and subsequent development of SBI. Effective healthcare and community prevention efforts targeted toward reducing risk of bacterial infections could prevent long-term hospitalizations, decrease health care expenditures, and reduce morbidity and mortality.

## Introduction

Bacterial infections due to injection drug use can occur locally at the site of injection or at distant sites through hematogenous spread. Localized infections in people who inject drugs (PWID) involving the skin and soft tissue (cellulitis, subcutaneous abscesses, and venous thrombophlebitis) rarely require hospitalization and are often managed without engagement in medical care [1]. Infections that spread hematogenously and seed distant body sites cause more severe infections, infectious complications, and often require prolonged hospitalizations for intravenous antibiotics and occasional surgical interventions [2, 3]. These severe bacterial infections (SBI) including bacteremia, endocarditis, osteomyelitis and central nervous system abscesses have been increasing in the last decade, mirroring the increase in prevalence of injection drug use [4–10]. Admission for severe bacterial infections in PWID, specifically endocarditis, is associated with sub-optimal treatment outcomes, high health care costs, and frequent readmission for re-infection [11–14].

Extensive studies have examined the injection risk behaviors associated with infectious transmission of blood-borne pathogens such as human immunodeficiency virus (HIV) and hepatitis C virus (HCV) [15–19], with interventions reducing drug equipment (syringes, needles, cookers, and cottons) sharing practice proving successful in reducing infection incidence. Bacterial infections have been less well studied. Bacterial skin and soft tissue infections (SSTI) are extremely common in PWID with an estimated annual incidence of 155,000 to 540,000 cases in the United States (U.S.), with the majority of these infections not interfacing with the healthcare system [20]. Despite most cases being managed in the community, SSTI remain one of the most common causes of hospital admission and emergency department visits among PWID [1], with infections caused by resistant bacterial such as methicillin-resistant *Staphylococcus aureus* seen disproportionately [21–24].

Prior studies have identified multiple risk factors for bacterial infections in PWID and include injection hygiene, injection frequency, route of administration, anatomic site of injection, polysubstance use—increased risk with opiate-stimulant combinations, as well as length of time injecting, homelessness, gender, sex work, and HIV [25–27]. Social-structural determinants of injection-related bacterial infections have been highlighted in the literature, including environmental constraints on the use of sterile water for drug preparation, how providing sachets of acidifier that were too large led directly to over-use of acidifiers and venous sclerosis, and how social and environmental conditions may lead to increased injecting-related skin and soft tissue damage [28–30]. Social-ecological models with respect to drug-related risk and harms emphasize the complex interplay between both behavioral and structural factors, with the need for a multi-level approach to harm reduction [31, 32].

Fentanyl has been a less well-studied potential risk factor for severe bacterial infection. Fentanyl-adulterated and/or fentanyl-substituted heroin integrated into the U.S. drug supply in the early 2010s and the vast majority of “heroin” tested positive for fentanyl by the end of the decade [33]. The introduction of synthetic opioids into the US has resulted in a significant increase in U.S. opioid overdose death rates [34]. Fentanyl, with its associated increased injection frequency and high concentration of cutting agents, has been implicated in altering injection drug use practices and subsequent risk of development of SBI [35, 36].

In the context of the current U.S. opioid epidemic, there remains a paucity of research with respect to identifying risk factors for the development of severe bacterial infections in PWID. In the following study, we present qualitative data examining injection and non-injection risk factors associated with bacterial infections among PWID hospitalized with severe bacterial infections.

## Methods

In this qualitative study, 15 participants (ages 27–57) who self-reported recent injection drug use and admitted to Bellevue Hospital with a SBI were recruited between August 2020 and June 2021 for individual interviews. Bellevue Hospital, the oldest public hospital in the United States, is an 844-bed acute care tertiary hospital located in New York City, New York. Participants were referred to study investigators for eligibility screening from the Infectious Diseases and/or Addiction Medicine inpatient consult services. Eligibility criteria included: reported history of recent injection drug use in the 90 days prior to admission; admitted to an acute care hospital with primary diagnosis of a severe bacterial infection; and ability to comprehend study procedures and provide informed consent. Severe bacterial infections were defined as skin and soft tissue infections requiring surgical intervention (e.g., abscess, necrotizing fasciitis), bacteremia, endocarditis, CNS infections or bone/joint infections (e.g., osteomyelitis, septic arthritis). Each participant was compensated $50 at the conclusion of the interview. All study activities were approved by the Institutional Review Board of New York University Langone Hospital and New York City Health and Hospitals. All participants provided written informed consent prior to being interviewed.

In depth, semi-structured interviews lasted approximately 60 min each. All interviews were conducted on inpatient medical or surgical wards. Interviews were conducted by a member of the research team, including a sociologist and a medical doctor not involved in the treatment of the patient. The interview format was flexible and consisted of open-ended questions that inquired about structural and behavioral domains potentially related to several bacterial infections. Topical domains addressed in the interviews included the following: sociodemographic characteristics, initiation into drug use and specifically injection drug use, current drug use and past trajectories (including patterns of escalation, concurrent or intermittent use of other substances), injection drug use practices, severe bacterial infection knowledge and perceptions of risk, stigma and drug withdrawal during hospitalization, self-treatment and prior hospitalizations related to bacterial infections, as well as structural and psychosocial factors. Interviewees were able to introduce or elaborate on topics of specific relevance to their experience.

Interviews were digitally audio-recorded and transcribed verbatim. Resulting transcripts were descriptively coded and the data analyzed. Themes were identified on the basis of topic and recurrent patterns highlighted throughout analysis of multiple participants’ accounts. Theoretical interpretations resulted from comparative analysis of the most commonly voiced themes and attempted to connect key themes across individual accounts. All participant names have been replaced with pseudonyms. In addition to thematic analysis, key variables were recorded via interview and abstraction of electronic medical record data related to current hospitalization in order to better identify the patient population under study.

## Results

The sociodemographic characteristics of the 15 study participants are listed in Table 1. The majority of participants had a primary diagnosis of native valve infective endocarditis (*n* = 10), with other primary diagnoses including prosthetic valve infective endocarditis (*n* = 1), spinal epidural abscess (*n* = 1) and complicated SSTI (*n* = 3). Thirteen participants had a positive HCV antibody serostatus, of these seven had a positive HCV qualitative RNA during admission. All participants were HIV negative. Six participants (40%) reported at least one prior hospitalization for SBI associated with injection drug use. A causative organism for current hospitalization was identified in thirteen participants and in ten the causative organism was Staphylococcus aureus [methicillin-sensitive *Staphylococcus aureus* (*n* = 5), methicillin-resistant *Staphylococcus aureus* (*n* = 5)]. A polymicrobial infection was identified in five participants. ICU level care was required in seven participants, mean length of ICU days during admission was 12.3 days (range 2–34). Surgical intervention was required in eleven participants and included mitral valve replacement (*n* = 3), aortic valve replacement (*n* = 1), and tricuspid valve vegetation debulking (*n* = 3). Mean days of parenteral antibiotic administration was 34.3 (SD = 17.8). Nine participants were discharged prior to time of final data analysis and among this group mean hospital length of stay was 33.9 days (SD = 24.9). Four participants left hospital against medical advice during course of treatment and one participant eloped prior to treatment completion and then returned to hospital the following day. Participants’ basic drug-use characteristics are summarized in Table 2.

**Table 1:** Participant sociodemographics (*N* = 15)

	*n* (%)
Mean age: 38.7 years (SD = 9.7)	
Age range	
20–29 years old	2 (13)
30–39 years old	9 (60)
40–49 years old	1 (7)
50–59 years old	3 (20)
Gender	
Male	12 (80)
Female	3 (20)
Race/ethnicity	
White	8 (53)
Hispanic/Latino	4 (27)
African American	3 (20)
Currently homeless	
Yes	8 (53)
Street	4 (27)
Shelter	4 (27)
No	7 (47)
Education level	
Some high school	9 (60)
High school graduate/GED	4 (27)
Some college	1 (7)
College graduate	1 (7)

**Table 2:** Participant drug-use characteristics (*N* = 15)

	Mean (SD)	*N*	%	Range
Age at first drug injection, years	25.8 (7.1)	15	–	18–43
Injection drug use, years	12.9 (9.1)	15	–	1–31
Number of days injected in 30 days prior to admission	26.3 (8.5)	15	–	2–30
Number of injections per day in 30 days prior to admission	11.0 (8.3)	15	–	4–30
Drugs injected in 30 days prior to admission				
Heroin	–	12	80	–
Fentanyl	–	4	27	–
Cocaine	–	9	60	–
Crack cocaine	–	1	7	–
Methamphetamine	–	2	13	–

Injection risk behaviors and participants’ perception of these risks with respect to SBI were highlighted during the interviews with resultant emergence of themes.

### Lack of education on risks of SBI

#### Basic understanding of prevention of blood-borne viral transmission (e.g., avoid needle sharing) but limited understanding of bacterial infection risks

Most participants described an understanding of the risk for HCV and HIV transmission associated with sharing needles and other injection paraphernalia but have limited knowledge related to SBI risk and prevention. The knowledge regarding injection-related viral transmission was learned from harm reduction programs where most participants at one time or another have regularly collected safe injection paraphernalia. In contrast, during their interactions with harm reduction services, participants reported learning “nothing or very little” in terms of injection related bacterial risk and how to prevent.

The place that taught me mostly about the needle use was the needle exchange program (NEP) […]

I learned about it through other peers [at the NEP] the fact that you can catch HIV by sharing needles (Martha, age 39, Latina, female)

They [NEP] told me to try to get a new needle as often as possible um change your cotton, things like that or like hold a lighter to it but I mean a lot of the people that I was around there like you know their syringes didn’t have numbers on them anymore so they [peers at NEP] didn’t know much (John, age 30, White, male)

This knowledge about safe injection practices was often passed from family members or other friends/acquaintances that injected drugs.

My mom was a heroin addict and I grew up in a neighbourhood where it was a lot of people using drugs and at that time it was the HIV epidemic and I know that you can get the virus by sharing needles and I don’t want to be a victim of that (Martha, age 39, Latina, female)

Understanding blood-borne viral transmission risk was also reinforced as a result of participants’ lived experiences, which subsequently would lead to avoidance of needle sharing practices.

The one time I did [share a syringe] I got Hepatitis C from it and it had to have been…really the one time I paid for that by getting Hepatitis C (Brandon, age 38, White, male)

I used to [share], not this year […] because I caught hep C back in 2015 and I just got rid of it 2019 (Victor, age 53, Black, male)

Participants without a prior history of hospitalization from injection-related bacterial infections reported anecdotal experiences of SBI from fellow injection drug users.

I have this one friend who almost lost his leg from a fucking staph infection but he wouldn’t go into the hospital you know so I kind of learned from him to go the hospital (Eli, age 30, White, male)

I heard a story not too long ago about a kid that had an infection […] but other than that I don’t really hear anybody talk about it (John, age 30, White, male)

Despite underlying knowledge of the potential risk and general avoidance in sharing drug injection paraphernalia, participants mostly reported a perception of minimal individual risk and a generalized lack of understanding regarding safe injection practices for prevention of SBI.

Ya I’ve known that [sharing needles is risky], I feel like that’s pretty well known but I feel like that’s the only thing (John, age 30, White, male)

I just didn’t give a fuck, just didn’t care. You feel like you’re invincible, that shit ain’t gonna happen to me, oh they just say that stuff blah blah blah until it happens and you don’t really think it’s going to happen to you (Eli, age 30, White, male)

#### Prior injection-related SBI hospitalizations with omission of harm reduction knowledge aimed at preventing SBI

Five participants (50%) reported at least one prior hospitalization for SBI associated with injection drug use. Three participants had a prior history of endocarditis (including one with nine prior documented episodes), and one of these participants had undergone valve replacement surgery prior to current admission. Each participant understood that prior SBI was directly related to injection drug use yet few participants were provided knowledge or resources for prevention of injection-related SBI during previous hospitalizations. When participants were asked what they had learned regarding harm reduction for SBI during prior hospitalizations, John (30, White, male) responded with a recurring answer: *“I didn’t really learn anything.”*

Of those participants that did report receiving harm reduction strategies for SBI, it was described as primarily limited to needle sharing and re-use.

I didn't want to get an infection this time around and I because of the past I had infections and I used, I never used the same needle twice and um I was surprised when I got an infection you know because I was trying to be very safe at least so I thought I was you know (Brandon, age 38, White, male)

Additionally, it was described by several participants that even though harm reduction knowledge may be present, priority of risk reduction strategies may be diminished as a consequence of drug high.

The thing is that sometimes you just don’t think it’s going to happen again you know, you think you’re so smart that you’re going to clean the syringes and you’re going to try to do the right thing but when you get high you forget all that and then you start doing the wrong things again (William, age 57, Latino, male)

### Engagement in high risk injection behaviors prior to hospitalization with SBI

#### Polysubstance use (e.g., speedball)

Three participants used heroin and/or fentanyl exclusively. Seven participants reported daily polysubstance use with concurrent injection of heroin/fentanyl and cocaine (“speedball”), as well as two participants reporting additional intermittent methamphetamine (as well as concurrent with heroin/fentanyl termed a “goofball”) and crack cocaine injection drug use.

Almost everybody I know won’t even do one [fentanyl] without the other [cocaine] and vice versa...I think it’s because the fentanyl is so strong it will just make you nod out and then you don’t enjoy it when you’re asleep for your high so then people want to be up and active and then you get addicted to the euphoria from the cocaine, so before you know it you have two addictions (John, age 30, White, male)

Fentanyl use, as well as concurrent opioid and stimulant use, was reported to lead to increased injection frequency due to a perceived shorter high, often in excess of 15 separate injection events per day.

I would prefer good heroin, but it’s hard to find because fentanyl bro it don’t hold you. You’ll be sick in like 2 or 3 hours, your withdrawals come on faster with fentanyl, with heroin it'll hold you longer…I compare it like cocaine and crack right, cocaine would last a long time and wouldn’t be as intense of a high, then crack came along and it’s like a minute long high and then you fiend for it. That’s how heroin was like the cocaine, fentanyl is like the crack (Eli, age 30, White, male)

#### Tissue damage: repeated penetration of skin with same syringe per injection episode and vein fishing

Participants repeatedly reported poor vein health as a result of their history of injection drug use. Consequently, most participants reported needing multiple injection attempts per injection episode (MIPIE) in order to achieve a successful injection. John (30, White, male) responded to difficulty attempting to find a vein with *“oh god sometimes it can take up to an hour, sometimes it takes me a while, sometimes 20, 30, or 40 [punctures of the skin].”* Brandon (38, White, male) with a prior history of SBI described using a clean needle for every injection to prevent future SBI, however due to difficulty finding a vein noted *“I didn't re-use I would usually throw them away, um I mean there were times when you know the thing is I don't have any good veins left and um I would have to like stick myself multiple times.”* Difficulty achieving a successful intravenous injection on the first attempt was common and resulted in reluctance to rotate locations once a reliable spot was found.

It could take 2 or 3 times, and other times I could be poking for hours you know, but usually on average 5 times, like I’ll find a vein that I like, that I know is gonna hit and then I’ll know that I know how to hit that vein and then I’ll wear that fucking thing out until I have to find a new vein you know (Eli, age 30, White, male)

Vein “fishing” (effort to find a viable vein to inject the drug dilution after having penetrated the skin with the needle) was repeatedly addressed by participants as they noted that although they may miss on the first injection attempt, they do not always completely retract the needle out of their tissue and would continue to partially retract and adjust the angle in an attempt to find a vein. Additionally, one participant reported seeking a larger gauge needle due to difficulty finding a vein.

Usually I try to get 27 gauge which is bigger, it seems like I have more trouble with the little smaller ones than I do with the big ones (Brandon, age 38, White, male)

Specific substances may play a role in accelerating tissue damage. Additional substances may be needed to prepare drugs for intravenous administration, which is an acid in the setting of intravenous crack use. The use of acidic substances will further damage the veins.

Multiple ways you could break it [Crack cocaine], you can crush it down or break it down with lemon juice, vinegar, any vinegar will work, Kool-Aid mix, sometimes I’ll just use a straight lime or a straight lemon. I’m sure [it was harsher on my veins], I’m sure that’s the reason that my neck veins are burnt out (John, age 30, White, male)

An additional common practice leading to damaged tissue was the self-treatment of perceived minor injection-related injuries.

Injection-related SSTI, particularly superficial uncomplicated abscesses, were common among participants. Several participants reported injection site abscesses within the preceding 4 weeks prior to admission for SBI. Seeking medical care for injection-related SSTI was rare and participants opted for self-treatment given perceived minimal risk, frequent occurrences, and favorable outcomes in the past.

I had a bump swell up on my arm, it hurt and I left it, it went down, it hardened and then one day I seen pus oozing out of it, so I went to the drug store and I got peroxide, I got band-aids, I got gauze, tape and every day I would clean it…I’d start squeezing it, moving it, rubbing it, so more could come out, I’d clean it, I’d bandage it, the next day I’d press it some more, so more would come out and then it dried up (Victor, age 53, Black, male)

I’d missed the injection spot and it would kind of puff up, but a lot of times what I would do is I would take a needle and puncture holes into the abscess and use a warm, wet towel and kind of squeeze the infection or whatever stuff out of it and that usually helped, actually it usually worked (Brandon, age 38, White, male)

John (30, White, male) had previously been hospitalized for necrotizing fasciitis requiring emergent surgical intervention. He reported self-treatment of superficial skin abscess prior to admission stating *“the time before it was horrible, my arm was leaking, it was huge and stunk, I let it go way farther than I ever should have and I didn’t realize how serious it was, so from that knowledge I got the first one and I lanced it myself and I kept an eye on it and I said if it got worse I would go to the doctor, the second a second head came away from that is when I thought ok I might be in trouble.”*

#### Drug diluting water and multipurpose use of water container

Participant responses varied on their source of drug diluting water, ranging from sterile water obtained at a needle exchange program or from purchased bottled water, to anything available.

Sometimes if I don’t have clean water ill pick up bottles, most of the time from a bottle on the street […] like I’ve literally used spit because I couldn’t find water to drop (John, age 30, White, male)

Usually I’ll like look in the trash can for it, for a half-filled bottle or a bottle with some little fucking bit just enough you know. I would say that or if I have a drink [I’ll use that], it’s never water if I have a drink (Eli, age 30, White, male)

Tap water (non-sterile) was also a frequently reported water source among participants and Brandon (38, White, male) demonstrated he was unaware of potential for bacterial presence in tap water *“ya it was always clean water we were always close to faucets to get clean water.”*

Risk of bacterial contamination in drug diluting water was not limited to its source. Participants regularly reported use of a long-term multipurpose drinking bottle for drinking water, drug diluting water, and water to clean the syringe after injection. Victor (53, Black, male) and Derek (49, Black, male) noted that their main source of drug diluting water was a purchased water bottle that they would use for drinking water, re-fill with tap water. Victor noted the bottle would last *“a week or more”* and Derek stated the bottle *“would last me maybe a couple of weeks.”*

#### Lack of hand and skin hygiene injection practices

Participants at times attempted to wash hands with soap and water, not the skin overlying potential injection site. Skin hygiene was further limited by limited access to running water and soap by those participants who were homeless. Some participants that visited needle exchanges received alcohol swabs as part of their drug kit although reported use was sporadic, even in those with prior personal experience of SBI.

I was using them [alcohol pads] a lot of times but a lot of times I wasn’t […] it was laziness, too high, in a rush, many reasons but they’re not good enough, you’re supposed to always use them (Victor, age 53, Black, male)

I didn't properly clean the spot before I injected and that's got to be it the thing is like I said is I'm a hard stick so I would have to go find different areas in my arm like I tried even my foot before you know and that can't be that sanitary (Brandon, age 38, White, male)

Several participants reported no prior knowledge regarding hand and skin hygiene prior to injection.

#### Injecting concentrated solution and without use of filter

Filter/cotton use was widespread among participants although some reported injecting without a filter at times.

Using blood to dilute highly concentrated drug solution was reported by Derek (49, Black, male), who reported using anywhere from 20 to 30+ bags of heroin per day in the 30 days preceding hospital admission. Prior to injection he would mix multiple bags of heroin with minimal water and then stated he *“would let the blood get in the drugs so that way it would dilute it just a little bit more then I’d shoot it.”*

#### Injection equipment: bacterial contamination, manipulation, re-use and network sharing

Needle contamination was reported in a variety of ways with both sterile syringe use and re-use of syringes. Placing the tip of the sterile syringe in the mouth prior to injection was common practice for Martha (39, Latina, female) who noted “after I draw the heroin up with the water. There’s space right, so I go like that and then as I’m pushing it up I’ll put the needle on my tongue just to taste when it gets to the very end. I’ll taste it and then I’ll stop to make sure I don’t lose too much, I don’t have any air and I don’t lose any of the heroin.”

Additional sources of bacterial contamination can potentially occur during storage and manipulation of the syringe. John (30, White, male) reported storing needles for re-use in his pocket and stated “I put the cap on but it comes off a bunch, it’ll poke me in the leg…if it’s not bent I would [use it again].” Victor (53, Black, male) discussed instances where he would need to manipulate the used syringe to improve its working condition*—*“sometimes you might drop the needle and you don’t have to drop it on the floor you can drop it on a piece of wood and it will bend the needle so when you try to poke your skin it’s not going to go clean, so now you’re trying to straighten the needle out, you would take the needle and scrub it and rub it on the lighter to make it sharp.”

Participants reported occasional syringe sharing during instances of syringe shortages while injecting in the company of close network members where perceived HCV and HIV status was known.

No there's been times where I didn't have a needle at all and I would share. Like I would never pick one up off the floor. I made sure like I knew the person and I knew that they didn't have HIV or something like that. But I didn't properly clean it the way it's supposed to be. I would just run water through it, make sure there was no blood in it, and then use it. (Elaine, age 32, White, female)

I ended up sharing with him [fellow known PWID] like I had been in a program with him and it’s always just a quick run through like “oh I don’t have nothing. You have anything? Nope don’t have nothing. Alright cool” and then people are very quick to share (John, age 30, White, male)

Sharing of other drug injection equipment, including cottons/filters, cookers, and drug diluting water and/or containers, was more commonly reported among participants and perceived as less risky. Eli (30, White, male) has a close injection network of approximately ten individuals and reported “we’ll all share the water we usually all have our own cookers, cottons, and syringes but sometimes there’s a person that doesn’t have their own cotton or something and they need it, usually we always share the water though.” Brandon (38, White, male) reported a high degree of caution, with preference to inject alone and denied sharing syringes and equipment. On further probing he noted that in the preceding six months leading up to admission he had not shared syringes but could recall sharing equipment including cookers, cottons, and drug diluting water with up to 5 individuals who were not close injection network members.

All participants reported re-using their own syringes at varying frequency and typically dependent on sterile syringe availability to the individual. Most described cleaning the needle after each use with either tap or bottled water, some noted that there were still visible blood products within the syringe prior to next use.

There were days where it was just one fucking syringe […] I wouldn’t go to the exchange I was registered to […] I just used the fucking same [syringe] I called it a nail, no fucking numbers on it you know? (Eli, age 30, White, male)

Cotton and cooker re-use was far more common among participants and often more prolonged than syringe re-use. Victor (53, Black, male) reported he “would use the same cooker for so long” despite going to the needle exchange program and receiving new equipment regularly, when asked to elaborate on why he would re-use his cooker he stated “I’ll have a clean cooker sitting right there and instead of me putting the dope inside the clean cooker ill use the old one, that doesn’t make any sense.” John (30, White, male) elaborated further on why he preferred to re-use his cooker and cotton by reporting that “there’s like leftover shit from the previous one in the cooker and the filter gets caked up so you want to save that stuff […] you take water and you put inside the cooker and you throw your cotton in there that you used the last couple of times you smash the cotton up and when you smash the cotton up you get the residue and pull it up, usually it’s not much of a hit but it might get you from being real sick to like able to get up and do something to get some money you know.”

Participants reported storing cottons wet for a prolonged time in drug dilution from previous injections for a “cotton shot” when they did not have access to drugs. Cotton shots, when not shared, are perceived by participants as a safe last resort source for drugs, unaware that warm, moist environments facilitate bacterial growth.

Oh ya, we all save our cottons, we all save our cottons […] usually I put two cookers and I back em so like you put one in the other you know what I’m saying and it creates the seal for the moist cotton (Eli, age 30, White, male)

### Structural factors and unstable drug injection environment

Often, participants reported that homelessness would lead to more careless injection drug use practices in the setting of an unstable drug injecting environment, pessimistic thoughts regarding future, and lack of available resources.

I was just really careful you know what I’m saying I would always rinse it out and try to get new ones from the store and what not and like, once I hit the streets bro everything changed my whole mentality changed (Eli, age 30, White, male)

When I first started I wanted all clean everything you know like everything just had to be perfect, I made sure I cleaned my arm like I did all those things at first even though I didn’t really know like I knew but nobody was like do this, but like I just knew from like when you go to the doctor’s office plus I do tattoos like I understand being clean but it’s just I don’t have the resources, I don’t have as much money, I’m not as stable, where I’m shooting up isn’t the same, it’s just everything is different and at the end of the day just getting the shot in is more important than everything else (John, age 30, White, male)

## Opioid withdrawal symptoms and impact on failure to complete treatment course

Opioid withdrawal symptoms while hospitalized for SBI were present among most participants. Several participants reported that even though they were experiencing withdrawal symptoms despite initiation of medication-assisted therapy (MAT) and had cravings for drug use, they planned to complete treatment course due to perceived severity of infection and response from medical providers.

One night I was in the hospital and I was like I wanted to go, I was kind of stirring around, I wanted to leave, so I was just like I want to get out and do it […] I explained to them I’m heroin sick but I’m also sick from something else, but they listened to me and they upped my dose (Victor, age 53, Black, male)

The majority of participants with prior hospital admission for SBI related to injection drug use admitted to a history of leaving against medical advice (AMA) or eloping prior to completion of medical treatment. Following cardiac valve replacement surgery, Elaine (32, White, female) reported that prior to initiation of MAT “I told them that I was going to do the Vivitrol shot. I told them that I was going to stay clean forever. I told them anything that they wanted to hear. And that's why I ran out before I was questioned, before they were able to do that for me, you know what I mean, so that way I could get high because that's what I wanted.” Eli (30, White, male) eloped during his current admission and later returned, he explained that he left because “well I’m fuckin sick and I don’t feel good and I don’t want to fuckin die like that so I want to get some bags you know what I’m saying like the stores are about to open I was going to go hit a store and go do what I had to do get some bags and play it by year and they were like we’ll give you more morphine you know and they gave me more morphine but it didn’t do fuckin shit, they gave it to me over a half an hour, I don’t know if it was a fuckin trick or whatever […] I got dressed, no one batted an eye, people looked at me, they didn’t say anything and I just walked down this hallway, took the elevator out and just left and then I got 5 bags and I did my bags throughout the day and then once I did the last bag I called 911 and I was like I’m going back to the hospital and then I came back.”

Inadequate treatment of opioid withdrawal symptoms resulted in several participants reporting intravenous drug use while hospitalized.

I got cotton fever in the hospital a couple of nights ago because I fuckin found some cottons in my fuckin jeans and tried to do it and I didn’t even get high off this shit its brutal (Eli, age 30, White, male)

I used to do drugs in the hospital, I would have my dealer come and visit me but they were maintaining me, they would give me either morphine or um Subutex. The Subutex worked well when I did it, but I still did heroin on top of it when I could (Brandon, age 38, White, male)

## An ecosocial theory understanding of SBI risk in PWID

**Fig. 1:**
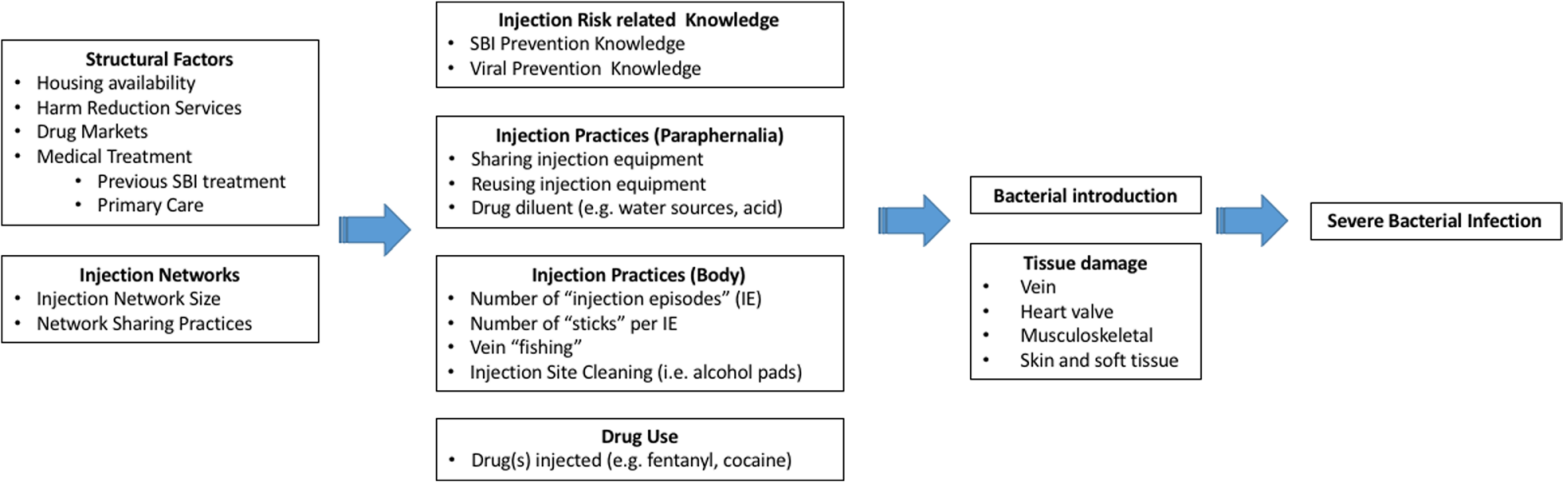
Socio-ecological model—multilevel risk factor associated with SBI

Based on our results we suggest an Ecosocial understanding of SBI risk (Fig. 1). As such, SBI risk is the result of a multi-level interplay between individuals and their social and physical environments in producing risk for negative health outcomes. We hypothesize that SBI risk among PWID is the result of structural factors and injection drug use networks directly impacting drug use, injection drug use practices, and harm reduction knowledge, which ultimately precipitates tissue damage and inoculation of bacteria into the host necessary for the development of SBI.

Our results indicate that multilevel risk factors interact to influence SBI risk. Firstly, we identified structural factors PWID face that could potentiate SBI risk, such as housing availability (i.e., homelessness), harm reduction services (e.g., syringe exchange program access), drug markets (e.g., fentanyl increasing frequency of injection), and medical treatment (e.g., previous SBI treatment could potentially reduce high risk injections, MAT could reduce frequency of drug injection). Secondly, the size of PWID’s injection networks might increase the risk of exposure to bacteria by increasing the number of individuals with whom potentially sharing drug diluting water, syringes and other injection equipment. How often PWID share drugs and injecting equipment within their networks, injection norms behaviors, and frequent engagement in high risk and unsterile practices, will also impact SBI risk. In turn, both structural and injection network variables affect multiple aspects of PWID’s drug use including: (a) knowledge of viral and SBI prevention; (b) paraphernalia-related injection practices (e.g., sharing injection equipment and water used to dissolve drugs); (c) injection practices at the body injection site (e.g., frequency of injection episodes, numbers of injections per injection episode, skin hygiene); and d) which drugs are injected (e.g., fentanyl, heroin, cocaine or a drug combination). The model highlights key aspects related to drug injection practices that may lead to development of SBI: unsafe drug paraphernalia use, which included sharing and reusing injection equipment as well as using non-sterile liquids to dilute drugs, high frequency injection drug use (i.e., increased number of injection events and number of “sticks” per injection event), vein “fishing” (searching the vein after needle puncture), and lack of injection site hygiene (e.g., using alcohol pads to clean injection site prior to puncture). The types of drugs PWID inject could also impact SBI risk, specifically increasing the frequency of injection events, for example, due to the shorter duration of the fentanyl high (versus heroin high) participants reported injecting more frequently throughout a given day.

Structural factors and injection networks have a direct impact on individuals’ injection-related knowledge and injection practices. All together these multilevel factors facilitate the introduction of bacteria into the body, where risk is amplified by repetitive tissue damage caused by repeated injection of drugs and potential injection of particulate matter and harmful compounds. Tissue damage is also inflicted by efforts to self-treat drug-related abscesses. Our analyses indicate that patients hospitalized with SBI have experienced different types tissue damage including: (a) localized (e.g., cutaneous and subcutaneous due to multiple injections, re-use of blunted syringes, and damaging substances including acidic diluents), (b) venous (e.g., repeated penetration leading to scarring, endothelial damage from damaging substances), and (c) distant (e.g., cardiac endothelial damage from particulate matter).

## Discussion

The above findings highlight the complexity of the injection drug use process and the potential social and physiological pathways leading to SBI. This study attempts to understand the multiple domains at the structural, network, and individual level that impact drug injection practices and provide context by which these factors predispose and lead to physiological tissue damage and the development of SBI among PWID. Our proposed Ecosocial understanding of SBI risk adds to pre-existing social-ecological models of drug-related harms by proposing pathways to tissue damage and ultimate development of SBI.

Cumulative damage to the skin and soft tissue at the site of injection increases susceptibility to infection. Tissue damage at sites distant from the injection event, including degenerative changes, areas of prior trauma and valvular endothelial damage associated with injection of particulate matter, likely increase the individuals risk of SBI. Underlying tissue damage at local and distant sites helps create an environment that is favorable for the adherence and proliferation of bacteria, ultimately leading to the development of SBI. High intensity injection drug use is likely associated with increased risk of SBI as Islam et al. demonstrated that reducing injection intensity is associated with decreased risk of invasive bacterial infections among high-frequency injection drug users [27]. Despite the known bacterial infection risk associated with intravenous drug use, few studies have attempted to link the epidemiological and physiological factors associated with SBI in PWID, our study highlights this interaction by the following:

Harm reduction knowledge and psychosocial vulnerabilities influence drug use and high-risk drug injection practices. Perceived likelihood of risk, severity, and susceptibility of SBI among PWID varies widely, and these beliefs can lead to risky injection practices in the context of withdrawal symptoms, drug injection network, and lived experiences. A repeated or temporary lapse in safe injection practices was a recurring theme, often in relation to the severity of addiction, feelings of hopelessness, and vulnerable social situation. Similarly, as reported by others, participants described putting on hold safe injection practices often when “sick” from opioid withdrawal [37]. Those challenging periods undermining PWID willingness to inject safely likely increased opportunities for bacterial introduction during the injection process.

Participants reported high rates of concurrent opioid and cocaine injection drug use (“speedball”). Fentanyl and cocaine were both reported to increase the frequency of injection. High frequency drug use is likely associated with increased cumulative tissue damage and multiplies the number of opportunities for bacterial introduction^.^ Furthermore, the short half-life of these substances may precipitate more frequent withdrawal episodes and therefore, as indicated above, lead to increased frequency of unsafe injection practices. MIPIE, repeated injection at a single body site, vein “fishing,” and use of larger gauge needles cause cumulative damage to skin and soft tissue that may increase SBI risk. MIPIE has been reported to increase the risk of HIV and HCV infections [37]. Increased risk of bacterial infections with “speedball” and other opiate-stimulant combinations has also been reported in the literature [38, 39]. In addition, cocaine may have a greater SBI risk compared to other street drugs as it can induce constriction of blood vessels, resulting in tissue damage secondary to inadequate blood flow, and may have greater propensity to cause injury to the myocardial surface [40, 41]. Additionally, crack cocaine is not readily soluble in water and is typically prepared using an organic acid, potentially furthering tissue damage and providing a favorable environment for bacteria [42]. One participant reported frequent injections of crack cocaine and over time this led to extreme difficulty finding a usable vein. Concurrent drug use in the same injection increases the likelihood of multiple different cutting agents being present, which may increase the odds of particulate matter entering the bloodstream and damaging the valvular endothelial cells. We posit that this repeated tissue damage both locally and at distant sites increases the risk of SBI among PWID. This direct impact of these external factors on and in the bodies of PWID is consistent with the notion of embodiment brought forward by Krieger. Embodiment refers to how humans literally incorporate, biologically, the world in which we live, including our societal and ecological circumstances [43, 44].

Lack of appropriate hygiene, storing of used (contaminated) syringes, re-using and sharing equipment, use of non-sterile drug diluting fluid, can provide opportunities for introduction of bacteria during the injection process. Socioeconomically disadvantaged participants frequently reported difficulties finding sterile water to use as a drug diluent and would often use discarded or re-used bottles as a source of water, increasing the risk of oral flora contamination of these water sources. Failure to adequately perform skin hygiene prior to injection and subsequent re-use of the needle could lead to the contamination of the syringe with skin flora. Storage of cottons/filters and cookers already exposed to wet material could provide an environment for bacteria to remain viable and proliferate. The re-use of this drug injection equipment not only exposes the PWID to bacteria [45] but potentially a higher inoculum than other possible pathways for bacterial introduction. This study indicates the need for widespread provision of harm reduction supplies to PWID including clean injecting equipment and sterile water.

Self-treatment of abscesses by PWID is common and may lead to the development of SBI. Localized SSTI as a result of injection practices was a recurring theme and perceived to be manageable without seeking medical attention. Perceptions regarding risk, anecdotal experience with self-treatment, as well as physical factors including one’s addiction may explain reluctance to seek medical care. Additionally, prior negative experiences within the context of inadequate opioid withdrawal management may also lead to reluctance to seek medical treatment [46–48].

Practice guidelines for uncomplicated skin abscesses following incision and drainage recommend antibiotic therapy to decrease the risk of infection [49]. Hygienic conditions and therapy are not easily available to the PWID who self-treat. Inadequate treatment and persistence of infection may precipitate hematogenous spread. Underlying tissue damage both locally and at distant sites would allow for adherence and propagation of bacteria. Increased medical management of uncomplicated SSTI among PWID would likely decrease rates of treatment failure and risk of progression to SBI. For participants with prior SBI hospitalization, adaptation of safer injection practices as a result of education and past experience were reported to occur yet did not lead to prevention of future SBI in this population. This may be related to limited knowledge and omission by healthcare providers surrounding the multiple potential amplifiers of bacterial infection risk during the injection process.

PWID admitted to hospital with SBI should be treated in a multidisciplinary manner with particular focus on avoidance of withdrawal symptoms to limit failure to complete treatment and potential high-risk behaviors while hospitalized. Participants reported using intravenous drugs while hospitalized and noted unsafe injection practices (e.g., needle re-use, using medically placed venous catheters) while doing so. Managing withdrawal symptoms in hospitalized PWID with SBI would ultimately decrease risky injection practices that may lead to severe complications, elopement or leaving the hospital against medical advice, as well as ensuring completion of treatment course [50].

The evolving opioid epidemic coupled with limited knowledge of potential risk factors and increasing incidence of SBI in PWID, provides a significant opportunity for intervention that may reduce morbidity and mortality in this vulnerable population. Harm reduction strategies targeting SBI will need to be comprehensive given multiple potential means of introducing bacteria into the process and fluid nature of the risk (during and between separate injection events). The typical medical professional offers minimal information (i.e., clean needle use, avoid needle sharing) for safe injection in the context of complex and varied behaviors. PWID interviewed in this study demonstrate what is likely widespread basic understanding of safe injection practices. In addition, PWID interviewed noted information about “safe” practices often travels via word of mouth rather than from medical professionals. PWID would benefit from pervasive messaging throughout the medical system, provided through a more complex and in depth understanding of the potential risks and prevention strategies.

Harm reduction messaging consistently acknowledges the importance of access to clean injection equipment in the prevention of blood-borne viral pathogens such as HIV and hepatitis C, however guidance on the prevention of bacterial infection is limited [51, 52]. Amending current harm reduction messaging is likely to be important, especially in the context of growing acceptance of observed consumptions sites [53], and potential opportunity at these facilities to identify PWID that may be at higher risk for bacterial infections as a result of their underlying tissue damage or injection practices. Interventions could include earlier medical evaluation for skin and soft tissue infection and training to improve sterile, and less risky, injection practices. Lastly, beyond injection supplies and prevention knowledge, the embodiment of harsh socio-ecological factors leading to SBIs (and other infections), calls for a forward acknowledgement and welcoming of PWID’s bodies in harm reduction services. As such, availability of food for those who are hungry, a place to rest for those who are tired, a nurse to attend to sickness and abscesses, clean clothing, and showers for those who cannot have access to them would go a long way in facilitating the hygienic conditions SBI prevention requires.

## Limitations

This study is limited by a small sample size of 15, predominantly male, White, and older than what may be representative of other PWID populations within NYC or the U.S. Potential bias in participant responses could have occurred given participants were recruited and interviewed in a medical setting, and a member of the research team conducting the interviews included a medical doctor. Convenient recruitment of participants may introduce bias and limits generalizability of the results. Future work should aim to validate our proposed theory with larger samples and increased diversity within the participants.

## Conclusion

Structural factors and injection drug use networks directly impact drug use, injection drug use practices, and harm reduction knowledge, ultimately resulting in tissue damage and inoculation of bacteria into the host and subsequent development of SBI. Despite perceived safe injection practices among PWID, limited practice of these behaviors and knowledge deficit on how to reduce their risk of drug-injection-related SBI was common. Effective healthcare and community prevention efforts targeted toward reducing risk of bacterial infections could prevent long-term hospitalizations, decrease health care expenditures, and reduce morbidity and mortality.

## Data Availability

The datasets generated during and/or analyzed during the current study are not publicly available due to them containing information that could compromise research participant privacy/consent but are available from the corresponding author on reasonable request.
